# Quality of sick child management by health extension workers: role of a complex improvement intervention

**DOI:** 10.1186/s12913-023-09131-1

**Published:** 2023-02-16

**Authors:** Dawit Wolde Daka, Muluemebet Abera Wordofa, Della Berhanu, Lars Åke Persson, Mirkuzie Woldie

**Affiliations:** 1 Faculty of Public Health, Department of Health Policy and Management, Jimma University, Jimma, Ethiopia; 2 Faculty of Public Health, Department of Population and Family Health, Jimma University, Jimma, Ethiopia; 3 Ethiopian Public Health Institute, Addis Ababa, Ethiopia; 4 The London School of Hygiene & Tropical Medicine, London, UK; 5 Fenot Project, University of British Columbia, School of Public Health and Population, Addis Ababa, Ethiopia

**Keywords:** Complex intervention, Quality of clinical care, Sick children, Health extension workers, Ethiopia

## Abstract

**Background:**

Despite the expansion of the Integrated Community Case Management services for childhood illness, quality and utilization of services have remained low. To address the problem, the Government of Ethiopia introduced a complex intervention that included community engagement, capacity building of health workers and enhanced district-level ownership of sick child management. We examined whether this complex intervention was associated with improved management of sick children by health extension workers.

**Methods:**

The study was conducted in four Ethiopian regions. A baseline survey was conducted in 26 intervention and 26 comparison districts from December 2016 to February 2017, followed by an end-line survey 24 months later. We observed health extension workers’ consultations of sick 2–59 months old children. The analysis has evaluated if children with pneumonia, diarrhoea and malnutrition were assessed, classified and treated according to guidelines, and included difference-in-difference analyses.

**Results:**

We observed 1325 consultations of sick children. At baseline, 86% of the sick children with cough in the intervention areas and 85% in comparison areas were assessed according to the guidelines, without any change at end-line associated with the intervention (difference-in-difference = -21%, *p* = 0.55). Sixty-two percent of children were assessed for dehydration at baseline in intervention and 47% in comparison areas, with no improvement associated with the intervention. Similarly, 87% of sick children in intervention and 91% in comparison areas were assessed for malnutrition, with no change over time associated with the intervention (difference-in-difference = 5%, *p* = 0.16). Appropriate pneumonia treatment with antibiotics declined and diarrhea treatment increased in both areas. Half of the malnourished children received ready-to-use therapeutic foods without any improvement associated with the intervention.

**Conclusion:**

The intervention was not associated with improved quality of the health extension workers’ management of sick children. The lack of association may be linked to low fidelity in the implementation of the intervention. Our findings suggest that training healthcare providers without continued clinical mentoring and support does not improve the quality of care. Community-based programs can be strengthened by ensuring high coverage and continued clinical mentorships, supportive supervision, and supply of medicines and other essential commodities.

**Trial registration number:**

ISRCTN12040912, retrospectively registered on 19/12/ 2017.

## Introduction

Pneumonia and diarrhoea remain major killers of young children, despite the existence of proven interventions [1, 2]. These two illnesses account for one-third of all under-five child deaths and a loss of two million young lives per year [3]. The burden of these health problems is also higher among malnourished children. Nutrition-related factors contribute to about 45% of these deaths [4, 5]. Although the incidence of pneumonia and diarrhoea has declined in the past few decades worldwide, these reductions are not uniform across regions, with sub-Saharan Africa and Southern Asia having the highest burden of morbidity [6–8].

The community-based management of suspected pneumonia using antibiotics, diarrhoea with Oral Rehydration Solution (ORS) and zinc, and malnutrition with ready-to-use therapeutic food plays a key role in providing prompt care to reduce mortality in low-resource settings [9–14]. Ethiopia established the program for Integrated Community Case Management (iCCM) of childhood illness in 2010, comprising the management of pneumonia, diarrhoea, malaria and acute malnutrition; referral of severe illnesses; and, counselling parents of sick children [15]. These services are provided by health extension workers at health posts as part of the Health Extension Program, which itself was introduced in 2003 [16].

Providers’ adhere to clinical guidelines in the assessment, classification and treatment of sick children is low in most low-and-middle income countries; indicating the missed opportunities to provide them with lifesaving interventions [17–19]. In Ethiopia, in the early implementation period, studies assessing iCCM indicated that the quality of these services was good [20]. The program had strong leadership and support from the government [15]. Appropriate managerial and clinical support also improved the health extension workers’ performance [21]. Over time, despite the expansion of the iCCM services, quality and utilization of services have remained low. Studies have reported that assessment, classification, and management of sick children need improvement [22, 23] and that the referral practices for severely sick children was weak [22, 24]. Lack of medicines, supplies, supportive supervision and mentorship, and a decay in the health workers' clinical knowledge have been identified as challenges facing the program [25–28].

Based on a scoping review and a qualitative study that analysed the barriers and facilitators of child health service utilization, the Government of Ethiopia introduced a complex intervention called Optimizing the Health Extension Program (OHEP) [29, 30]. This intervention was implemented in four regions of Ethiopia (Oromia, Amhara, Tigray and Southern Nations, Nationalities, and Peoples region) from 2017 to end of 2018. OHEP aimed to increase the utilization of child health services through three main strategies: engaging communities, strengthening the capacity of the health extension workers to improve the quality of care they provide, and increasing the district-level accountability and ownerships for child health services [31]. Improved quality of care by health extension workers was an intermediate outcome of strengthening their capacity. The intervention was implemented by UNICEF, Last 10 Kilometers/John Snow, Inc., Save the Children and Program for Appropriate Technology in Health (PATH) in collaboration with the Ministry of Health and Regional Health Bureaus. The evaluation of the effectiveness of the OHEP intervention on the primary outcome, the utilisation of services for sick under-five children, has been reported and revealed that there was no effect of the intervention on the utilisation of services for sick children aged 2–59 months [32].

This study aimed to examine if at all there was any association between the intervention and the quality of assessment, classification and treatment of sick under-five children by health extension workers at health posts, which we considered to be in the pathway to increase service utilization for sick under-five children. This was done by conducting observations of health workers' management of sick children, which was then compared to the iCCM clinical guidelines. We focused on three major child health problems in this analysis, i.e., pneumonia, diarrhoea, and malnutrition.

The World Health Organization Health Facility Survey Tool was used to assess the quality of care delivered to sick children attending primary-level outpatient health facilities using the Integrated Management of Childhood Illness (IMCI) clinical guidelines as a best practice. This tool measures how well heath care workers’ asses, classify and treat under five children and also how well they council the caretakers. This is done by conducting observation of health workers' management of sick children, exit interview with caretakers, re-examination of the children by a health worker that serves as a “gold standard”, and assessing availability of equipment and supplies. In this paper, we have used information from the clinical observation sick-child module. The tool was validated and has been applied in various low-and-middle-income countries [33, 34].

## Methods

### Study design and setting

**Fig. 1:**
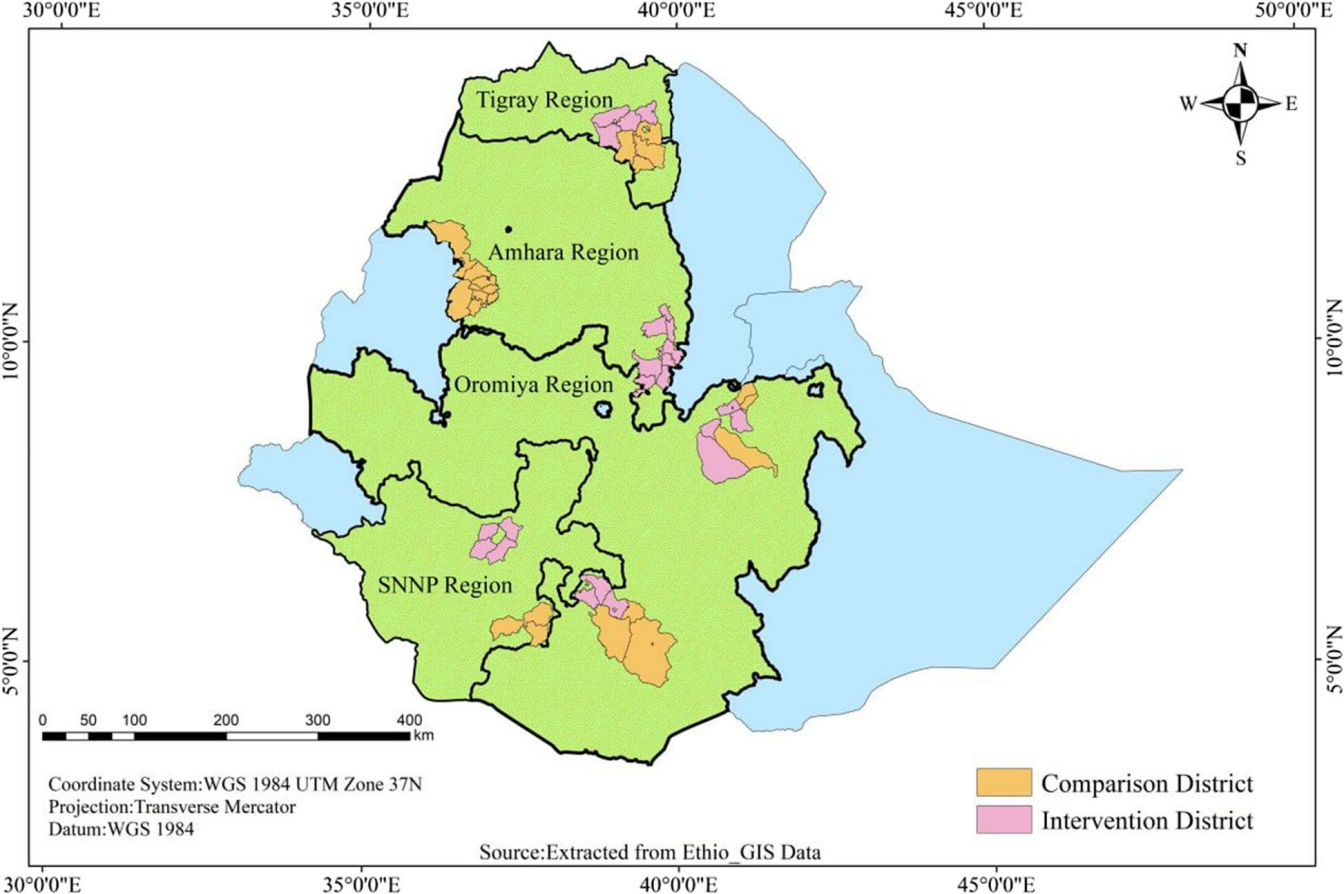
Study area map

Baseline and end-line surveys in the intervention and comparison areas were conducted from December 2016 to February 2017 and from December 2018 to February 2019, respectively. The study was conducted in 52 districts across the four most populous regions of Ethiopia: Oromia, Amhara, Tigray and Southern Nations Nationalities and Peoples’ regions. Twenty-six of these districts were intervention districts, where the complex intervention was implemented for two years; from 2017 to end of 2018 (Fig. 1). The intervention districts were selected by the OHEP implementing partners and the Ministry of Health for having poor primary health service utilization indicators for under five children. All the district health offices, health centers, health posts and community members within the districts were targeted by the program. The 26 comparison districts were identified by regional health authorities based on their similarity in population size and key maternal and child health indicators. The study districts were also comparable with respect to the health service coverage and other health system characteristics [32]. The intervention protocol has been published [31] and a summary description of OHEP strategies to improve sick child quality of care are presented in Table 1.

**Table 1:** Description of Optimizing Health Extension Program sick child health services quality improvement strategies

The Optimizing Health Extension Program comprised of three strategies. These were demand creation, capacity building, and district level accountability and ownership of child health services. Of these, the capacity-building strategy primarily focused on improving the quality of care for sick children. The strategy included gap filling training for health extension workers with no iCCM training, supportive supervision, and performance review clinical mentoring meetings. Quarterly supportive supervision was planned to be conducted jointly by staff from the district health system and implementing partners using standard checklists. The existing biannual performance review and clinical mentoring meetings were to be supported to take place by trained experts over the course of two days in the presence of health extension workers and their supervisors from their respective health centers and district-level health offices. The meetings aimed to review the six months integrated community case management performance of the health extension workers, analyze gaps, hold discussions on the identified gaps, provide mentoring, and develop action plans. Where missing, job aids were to be distributed which included registers, chart booklets (clinical guidelines), and health education aids such as posters and family health cards. Backpacks were to be introduced for use by the health extension workers to carry medicines and registers during their home-to-home services. As part of the district level accountability and ownership of child health services, implementing partners also aimed to advocate for: the integration of child health service indicators in the districts’ health planning and management system; use of ambulances for referral of very sick children; and, strengthening the linkages between health centers and health posts. The linkages and integration were intended to enable the health centers to provide a regular supportive supervision, as well as essential drugs and supplies to their catchment health posts. Although medicines were not directly supplied by the program under this strategy, activities were also planned to strengthen medicine supply to the health posts through the existing government system.	

### Study participants and selection procedure

A list of enumeration areas in the 52 districts were identified from the 2007 Ethiopian housing and population census. Two hundred clusters (enumeration areas) were randomly selected proportional to the population size of the districts. The health posts in the selected clusters were included in the study. The sick children who visited the selected health posts to seek care from health extension workers at time of the data collection were included.

A total of 800 sick child consultations were planned to be observed across intervention and comparison areas at baseline and again at end-line, with an average of four sick child observations per health post. This sample size would allow us to detect a 10–15 percentage point difference in the quality of health care provided by health extension workers between intervention and comparison areas at baseline and end-line, with a power of 80%. Given the low number of children that seek care at health posts, to ensure that we achieved this sample size, data collectors in collaboration with kebele (village) administrators mobilized families to bring their sick children to the health posts. This ensured that data collectors did not stay in the field for an extended amount of time. A similar approach of mobilization was employed both at baseline and end-line surveys.

### Measurements

We used a structured observation questionnaire to collect data. The questions and content of the tool were developed based on the World Health Organization tool to evaluate the quality of care delivered to sick children attending outpatient facilities [35] and the iCCM guideline developed by the Federal Ministry of Health in Ethiopia [36].

At baseline and end-line surveys, the questionnaire included the following sections: complaints, assessments, classification, treatment, counselling and referral. The observation questionnaire also assessed the service experience of caretakers at health posts and background information about the health extension workers. The questionnaire was translated into three local languages (Afan Oromo, Amharic and Tigrigna) and uploaded on tablets (CSPro 6.3 at baseline and CSPro 7.1 at end-line) for data collection. The questionnaire was pilot tested and revised.

Data were collected by experienced and trained data collectors who were health professionals and had received refresher training on the iCCM guidelines for the clinical management of sick under-five children. The data collectors and supervisors were trained for 10 days and also participated in the pilot test. The training included a detailed review of the questionnaire, data collection techniques, field procedures and research ethics. Data collectors and field supervisors were blinded to whether a district was an intervention or comparison area.

The overall data collection process was strictly monitored by supervisors at field level who were health professionals with a bachelor’s degree and above. In addition, a data manager performed daily data quality checks and provided feedback to the field-level team on identified errors. A similar approach of data collection was undertaken both at baseline and end-line surveys.

Children 2–59 months, who were considered sick and brought to the health post by their parents, were included in the study. Observation of sick child consultations with health extension workers was conducted and caregivers were interviewed after completion of the consultation.

### Data processing and analysis

The completeness and consistency of data were checked. The data analysis was conducted using Stata V.16 (StataCorp LLC, College Station, Texas, USA). First, the characteristics of study participants were analysed and presented using means, frequencies, and proportions. We conducted chi-square tests for all categorical variables and t-test for one continuous variable (average service year of HEWs) to assess if any differences existed between intervention and comparison areas over time. Second, the sick child complaints were analysed and presented. Third, the main outcome variables (appropriateness of sick child health care or quality of care) were analysed. Quality of care referred to the appropriate assessment, classification, and treatment of sick children as compared to the iCCM algorithm. In this study the appropriate management of common childhood illnesses, i.e., pneumonia, diarrhoea, malnutrition, were analysed (Table 2). The analysis was done for intervention and comparison areas at baseline and end-line.

**Table 2:** Definitions of outcome indicators

Indicators	Description and definition
Assessments	
Proportion of sick children assessed for cough/pneumonia	[Sick children assessed for cough through counting respiratory rate in 1 min divided by all sick children reported to have a cough by their caregiver or parent] multiplied by 100
Proportion of sick children assessed for dehydration/diarrhoea	[Sick children assessed for dehydration by pinching and checking whether abdomen of skin goes back slowly or very slowly divided by all sick children reported to have diarrhoea by their caregiver or parent] multiplied by 100
Proportion of sick children assessed for acute malnutrition using mid-upper arm circumference tape	[Sick children 6–59 months assessed for acute malnutrition using mid-upper arm circumference tape divided by all sick children 6–59 months old reported to have an illness by their caregiver or parent] multiplied by 100
Classification	
Proportion of sick children with appropriate classification of pneumonia	[Sick children appropriately classified for pneumonia divided by all children labelled with fast breathing and normal breathing] multiplied by 100
Proportion of sick children with appropriate classification of acute malnutrition	[Sick children classified as having acute malnutrition divided by all children with mid-upper arm circumference tape measurements < 11.9 cm] multiplied by 100
Treatment	
Proportion of sick children appropriately treated with antibiotics for pneumonia	[Sick children appropriately treated with antibiotics (correct dose, frequency and duration) divided by all children classified with pneumonia] multiplied by 100
Proportion of sick children appropriately treated for dehydration/diarrhoea	[Sick children appropriately treated with ORS and Zinc (correct dose, frequency and duration) divided by all children classified as having diarrhoea with dehydration] multiplied by 100
Proportion of sick children appropriately treated with ready-to-use therapeutic food	[Sick children appropriately treated with ready-to-use therapeutic food (correct dose, frequency and duration) divided by all children classified with acute malnutrition] multiplied by 100

The complex-intervention was the main independent variable for the observed changes. Difference-in-difference analyses was undertaken ([(I_E_-I_B_)-(C_E_-C_B_)]; where I was intervention group, C comparison group, E end-line and B baseline) to determine the association between the intervention and the management of suspected pneumonia, diarrhoea and malnutrition by the health extension workers. The key explanatory variable for the outcomes of this study was whether the sick child lived in the OHEP intervention area or lived in the comparison area. A model was created that included an interaction term for the timing of the survey (baseline or end line) and the survey area (intervention or comparison area). This model allowed for the calculation of the odds of appropriate assessment, classification or treatment of sick under-five children for the three illnesses separately, in intervention areas as compared with comparison areas, accounting for any differences between the areas at baseline, with adjustment for the cluster sampling and identified confounding factors from the Chi-square and t-test. Logistic regression model was fitted to assess if there was any difference between intervention and comparison areas over time. *P*-value < 0.05 and 95% CI were applied. Stata svy commands were used to adjust for clustering at the health post level.

## Results

**Fig. 2:**
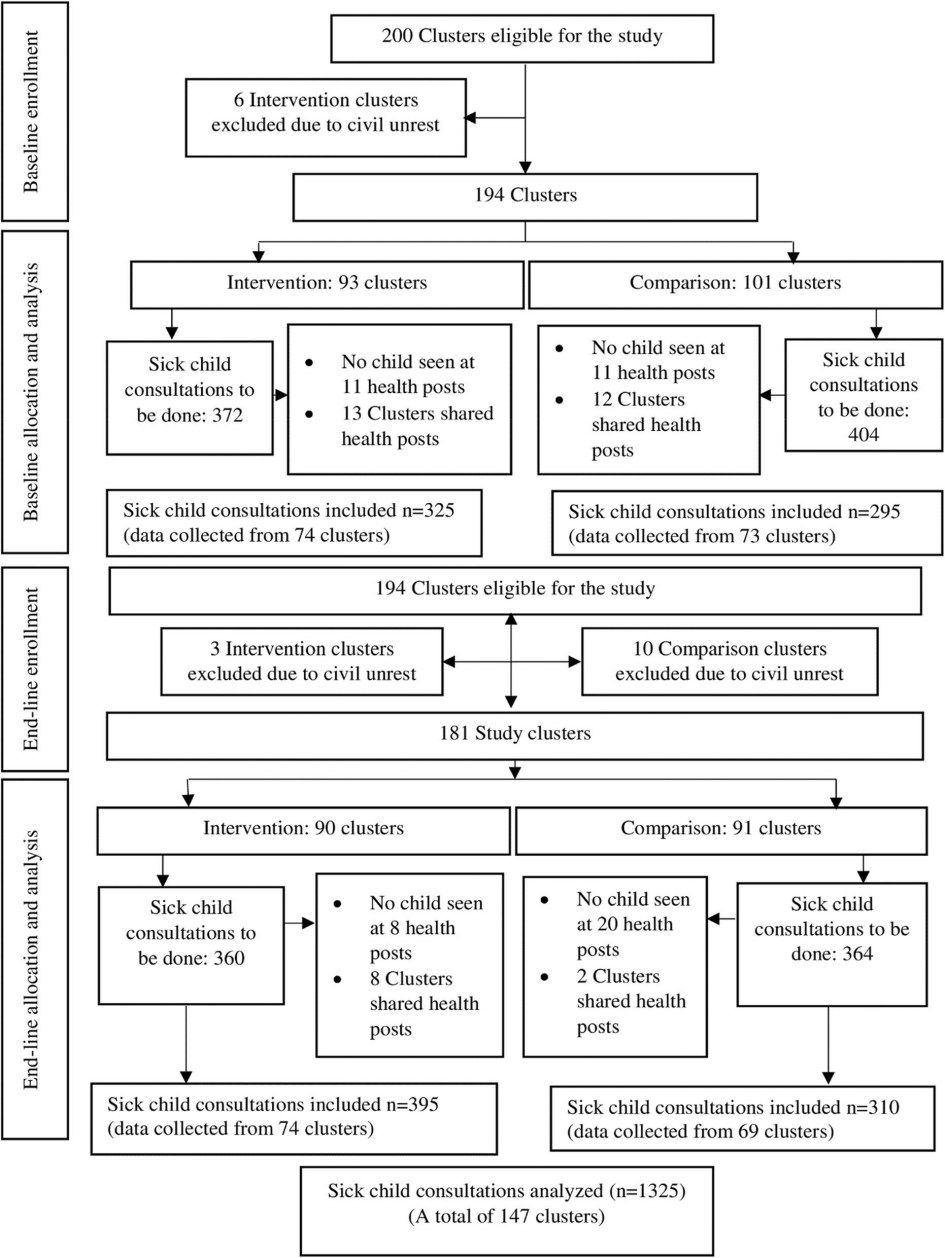
Study flow diagram

Of the planned 200 eligible clusters, data were not gathered from six intervention clusters at baseline due to civil unrest. At the end-line survey, another three intervention clusters and ten comparison clusters were excluded due to civil unrest. A total of 620 sick child consultations at baseline (325 intervention, 295 comparison) and 705 consultations at end-line (395 intervention, 310 comparison) were observed across 147 and 143 health posts, respectively (Fig. 2). Some of the clusters shared a health post and in other clusters data were not gathered since no sick child appeared at the health posts despite mobilization efforts. On average, four sick child consultations at the baseline survey and five sick child consultations at the end-line survey were observed per health post. The median duration of a sick child consultation was 20 min (IQR 12) at baseline and 20 min (IQR 16) at end-line. A description of the actual implementation of the OHEP quality of care strategies based on our published process evaluation is provided Table 3.

**Table 3:** The implementation processes of Optimizing Health Extension Program sick child health services quality improvement strategies

The Optimizing Health Extension Program implementation was fully operational from 2017 to end of 2018. Many of the interventions were delayed from the planned period and in one-third of the districts the implementation activities were interrupted for about 4 months because of administrative reasons or civil unrest. Overall, the process evaluation revealed that only few of the intervention packages had high implementation fidelity. The complexity of the intervention itself, weak support from the district health offices partly due to competing priorities, and irregular supervisions of the health extension workers were the main challenges of implementation. More specifically, from the strategies aimed at improving quality of care for sick children, the training of health extension workers in integrated community case management (iCCM), and provision of supportive supervision for health posts were implemented with a median district fidelity of less than 50%. In contrast, the performance review clinical mentoring meetings were implemented with higher fidelity in the period from 2017 to 2018 with a median district fidelity of 75% (IQR: 35–100). Regarding job aids and tools, the provision of backpacks and registration books for health extension workers had a median district fidelity of 100% (IQR: 0–100). The provision of chart booklet to the health extension workers had a median district fidelity of 62% (IQR: 0–100). The distribution of iCCM registration books for 2–59 months old children and chart booklets (clinical guidelines) were not implemented in eight districts where no budget was allocated for this activity [37].	

### Characteristics of sick children and health extension workers

The sex distribution of examined sick children was similar in intervention and comparison districts at baseline and end-line. At baseline, the age distribution differed between intervention and comparison areas, whereas at end-line it was similar. The distribution of sick children by region differed at end-line in both areas. In all study areas at baseline and end-line the major complaint reported by caretakers were respiratory problems followed by diarrhoea.

At baseline and end-line, no difference was observed in the proportion of sick children managed by Level-3 (certificate) and Level-4 (diploma) health extension workers in the intervention and comparison areas. The health extension workers who assessed children had similar number of years of experience at baseline and end-line in both areas. Most sick child consultations across areas were done by iCCM-trained health extension workers both at baseline and end-line.

At the baseline and end-line surveys in intervention and comparison areas, approximately seven in ten of the sick children were managed by health extension workers who had been supervised by health centre or district health office staff in the last three months. At baseline in both areas, less than half of the sick children were managed by health extension workers who had participated in performance reviews and clinical mentoring meetings in the six months prior to the survey. At end-line, there was an increase in both intervention and comparison areas. Nine out of ten sick children were managed by health extension workers that used the iCCM chart booklet and iCCM registration books in the intervention and comparison districts at baseline and end-line surveys (Table 4).

**Table 4:** Characteristics of sick children and health extension workers at baseline (December 2016 to February 2017) and end-line (December 2018 to February 2019) in the intervention and comparison areas

Variables	Baseline survey					End-line survey				
	Intervention (*N* = 325)		Comparison (*N* = 295)		*P*-value***	Intervention (*N* = 395)		Comparison (*N* = 310)		*P*-value****
	n	% (95% CI)	n	% (95% CI)		n	% (95% CI)	n	% (95% CI)	
Sex										
Boys	173	53 (48,59)	164	56 (50,61)	0.61	206	52 (47,57)	142	46 (40,51)	0.11
Girls	152	47 (41,52)	131	44 (39,50)		189	48 (43,53)	168	54 (49,60)	
Age in months										
2–11	126	39 (34,44)	77	26 (21,31)	0.02	128	32 (28,37)	106	34 (29,40)	0.91
12–23	94	29 (24,34)	96	32 (27,38)		121	31 (26,35)	89	29 (24,34)	
24–35	49	15 (11,19)	46	16 (12,20)		66	17 (13,21)	53	17 (13,22)	
36–47	34	10 (7,14)	41	14 (10,18)		46	12 (9,15)	40	13 (9,17)	
48–59	22	7( 4,10)	35	12 (8,16)		34	9 (6,12)	22	7 (5,10)	
Region										
Amhara	157	48 (43,54)	108	37 (31,42)	0.11	183	46 (41,51)	85	27 (23,33)	0.02
Oromia	76	23 (19,28)	121	41 (35,47)		119	30 (26,35)	151	49 (43,54)	
Tigray	32	10 (7,13)	36	12 (9,16)		58	15 (11,18)	66	21 (17,26)	
Southern Nations, Nationalities and People	60	18 (14,23)	30	10 (7,14)		35	9 (6,12)	8	3 (1,5)	
Complaints/ symptoms^a^										
Respiratory complaints	179	55 (50,60)	180	61 (55,66)	0.43	253	64 (59,69)	196	63 (58,68)	0.35
Diarrhoea	129	40 (34,45)	95	32 (27,38)		168	42 (38,48)	154	50 (44,55)	
Fever	87	27 (22,32)	77	26 (21,31)		80	20 (16,24)	58	19 (15,23)	
Vomiting	46	14 (11,18)	38	13 (9,17)		49	12 (9,16)	42	13 (10,18)	
Ear problem	16	5 (3,8)	21	7 (5,10)		20	5 (3,8)	13	4 (2,7)	
Other	22	7 (4,10)	18	6 (4,9)		50	13 (10,16)	26	8 (6,12)	
Average service experience (in years) of HEWs (Mean ± SD)	6.93	3.67	6.65	3.75	0.52¶	7.65	4.67	6.84	4.40	0.49¶¶
Proportion of children managed by^b^										
Level-3 HEWs	209	64 (59,69)	216	73(68,78)	0.25	217	55 (50,61)	148	48(42,53)	0.40
Level-4 HEWs	116	36 (31,41)	79	27 (22,32)		178	45 (40,50)	162	52 (47,58)	
Proportion of sick children managed by HEWs:										
For whom living house is provided	129	40 (34,45)	112	38 (33,44)	0.84	115	29 (25,34)	75	24 (20,29)	0.61
Proportion of sick children managed by HEWs:	325		295			395		310		
Trained in iCCM	297	91 (88,94)	264	89 (86,93)	0.69	325	82 (78,86)	262	84 (80,88)	0.73
That have been supervised in the past 3 months	249	77 (72, 81)	211	71 (66,76)	0.49	295	75 (70,79)	206	66 (61,71)	0.30
That participated in PRCMM^c^	150	46 (41,52)	133	45 (39,51)	0.90	299	76 (71,80)	203	65 (60,71)	0.19
That used chart booklet during consultations^d^	305	94 (91,96)	263	89 (85,92)	0.24	344	87 (83,90)	284	92 (88,94)	0.33
That used iCCM registration book during consultation	310	95 (93,97)	268	91 (87,94)	0.21	366	93 (90,95)	291	94 (91,96)	0.74

*CI* Confidence interval, *PRCMM* Performance review clinical mentoring meeting, *HEWs* Health extension workers, *iCCM* integrated community case management, *SD* Standard deviation

^a^A child may have more than one complaint; thus, the sum of the numbers in the individual columns be more than the total number of observed children

^b^Health extension workers (HEW) level of training categories: Level-3 HEWs are those who graduated with a certificate after a one-year pre-service trainings on the 17 packages of Health Extension Program. Level-4 HEWs are those who graduated with diploma after one-year additional education at a technical college

^c^Performance Review Clinical Mentoring Meeting (PRCMM) is a two-day performance review meeting in which the iCCM register are examined in day one, and clinical mentoring occurs on day two. Health workers trained as trainers facilitate PRCMM for 20–24 health extension workers. This event usually takes place in a meeting hall at a central town. Supervisors and peer-HEWs review the registers for consistency, completeness and caseloads (observed versus expected). It usually occurs biannually

^d^Chart booklet is a guideline or job aid that supports health extension workers in the assessment and management of sick children including referral and follow-up

^***^P-value for the difference between intervention and comparison groups at baseline using chi-squared test (significance level at *P* < 0.05)

^****^P-value for the difference between intervention and comparison at end-line using chi-squared test (significance level at *P* < 0.05)

^¶^*P*-value for difference between intervention and comparison groups at baseline using t-test (significance level at *P* < 0.05)

^¶¶^*P*-value for difference between intervention and comparison groups at end-line using t-test (significance level at *P* < 0.05)

### Assessment

As recommended by the Ethiopian iCCM guidelines, the health extension workers assessed pneumonia by counting breath for one minute for children with a respiratory complaint in 86% of the sick children in the intervention areas at baseline. This proportion decreased to 74% at end-line. In comparison areas, at baseline the health extension workers counted breaths in 85% of children with a respiratory complaint. This increased to 94% at end-line (difference-in-difference = -21%, *p* = 0.55).

At baseline in the intervention districts, the health extension workers assessed dehydration by pinching abdominal skin in 62% of sick children with diarrhoea. This assessment decreased to 42% at end-line. In comparison areas, 47% of sick children with diarrhoea were assessed for dehydration at baseline and 53% at end-line (difference-in-difference = -26%, *p* = 0.01).

The health extension workers had assessed acute malnutrition using Mid-Upper-Arm Circumference (MUAC) tape in over 80% of sick children in intervention and comparison areas at baseline and end-line surveys (difference-in-difference = 5%, *p* = 0.16) (Table 5).

**Table 5:** Proportion of appropriately assessed sick children at baseline (December 2016 to February 2017) and end-line (December 2018 to February 2019) in intervention and comparison areas

Indicators	Baseline survey					End-line survey					Difference-in-differences^a^ (%)	*P*-value†
	Intervention (*N* = 325)		Comparison (*N* = 295)		Difference (%)	Intervention (*N* = 395)		Comparison (*N* = 310)		Difference (%)		
	n	% (95% CI)	n	% (95% CI)		n	% (95% CI)	n	% (95% CI)			
**Pneumonia:** proportion of sick children with reported cough assessed by^b^-												
Counting breaths in 1 min	137	86(80,91)	140	85(79,90)	1	151	74(67,79)	138	94(89,97)	-20	-21	0.55
**Diarrhoea:** proportion of sick children with reported diarrhoea^c^-												
Assessed for dehydration by pinching and checking whether skin of abdomen goes back slowly or very slowly	79	62(53,70)	44	47(37,57)	15	58	42(34,50)	75	53(45,61)	-11	-26	0.01
**Acute malnutrition:** proportion of sick children assessed for acute malnutrition^d^-												
Using MUAC tape	236	87(83,91)	248	91(88,94)	-4	295	83(79,87)	233	82(77,86)	1	5	0.16

*CI* Confidence interval, *MUAC* Mid-upper arm circumference

^a^Difference-in-differences: the difference in the proportion between the intervention and comparison areas at end-line subtracted by the difference in proportion between intervention and comparison areas at baseline

^b^Denominator: Baseline (intervention = 159, comparison = 165); End-line (intervention = 205, comparison = 147)

^c^Denominator: Baseline (intervention = 128, comparison = 94); End-line (intervention = 138, comparison = 141)

^d^Denominator: Baseline (intervention = 270, comparison = 273); End-line (intervention = 354, comparison = 284)

^†^*P*-value obtained from the logistic regression model for the difference-in-difference analysis (for the difference between intervention and comparison groups over time). *P*-value was adjusted for clustering and age

### Classification

At baseline, the health extension workers appropriately classified the complaints and findings as suspected pneumonia in 86% (intervention area) and 85% (comparison area) of sick children with cough and fast breathing. At end-line, this performance decreased in the intervention (60%) and comparison (54%) areas (difference-in-difference analysis = 5%, *p* = 0.88). Health extension workers appropriately classified acute malnutrition in over 90% sick children with a MUAC of < 11.9 cm in intervention and comparison areas at both baseline and end-line surveys (difference-in-difference = 7%, *p* = 0.09). Similar data were not available for diarrhoea since the health extension workers did not record the results of skin pinch test (Table 6).

**Table 6:** Proportion of appropriately classified sick children at baseline (December 2016 to February 2017) and end line (December 2018 to February 2019) in intervention and comparison areas

Indicators	Baseline survey					End line survey					Difference-in-differences^a^ (%)	*P*-value†
	Intervention		Comparison		Difference (%)	Intervention		Comparison		Difference (%)		
	n	% (95% CI)	n	% (95% CI)		n	% (95% CI)	n	% (95% CI)			
**Pneumonia:** proportion of sick children-												
With respiratory complaints appropriately classified for pneumonia^b^	119	87(80,92)	120	86(79,91)	1	49	60(49,70)	35	54(42,66)	6	5	0.88
**Acute malnutrition:** proportion of children-												
With complaints appropriately classified for acute malnutrition^c^	232	93(89,96)	245	97(94,98)	-4	271	95(92,97)	200	92(88,95)	3	7	0.09

^a^Difference-in-differences: the difference in the proportion between the intervention and comparison areas at the end line subtracted by the difference in intervention and comparison area at the baseline

^b^Denominator: Baseline (intervention = 137, comparison = 140); End line (intervention = 82, comparison = 65)

^c^Denominator: Baseline (intervention = 249, comparison = 253); End line (intervention = 285, comparison = 217)

^†^*P*-value obtained from the logistic regression model for the difference-in-difference analysis (for the difference between intervention and comparison groups over time). *P*-value was adjusted for clustering and age

### Treatment

The proportion of children classified as having pneumonia that were appropriately treated with antibiotics slightly decreased from 60 to 54% in the intervention areas, while comparison areas showed a much greater decline from 92 to 41% (difference-in-difference analysis = 44%, *p* = 0.05). Appropriate treatment for children classified as having diarrhoea with ORS and zinc increased from 65% in the intervention areas at baseline to 82% at end-line. It also increased in comparison areas from 52% at baseline to 70% at end-line (difference-in-difference = -1%, *p* = 0.74). Forty percent of children classified as having malnutrition received ready-to-use therapeutic foods at baseline and 50% at end-line. In comparison areas, the corresponding figures were 70% at baseline and 33% at end-line (difference-in-difference 47%, *p* = 0.44) (Table 7).

**Table 7:** Proportion of appropriately treated sick children at baseline (December 2016 to February 2017) and end-line (December 2018 to February 2019) in intervention and comparison areas

Indicators	Baseline survey					End-line survey					Difference-in-differences^a^ (%)	*P*-value†
	Intervention		Comparison		Difference (%)	Intervention		Comparison		Difference (%)		
	n	% (95% CI)	n	% (95% CI)		n	% (95% CI)	n	% (95% CI)			
**Pneumonia:** proportion of sick children-												
Appropriately treated for pneumonia^b^	26	60(45,74)	23	92(76,99)	-32	30	53(40,65)	16	41(26,57)	12	44	0.05
**Diarrhoea:** proportion of sick children-												
With diarrhoea classification appropriately treated with ORS and zinc^c^	77	65(56,73)	43	52(42,63)	13	95	82(74,88)	77	70(61,78)	12	-1	0.74
**Acute malnutrition:** proportion of children-												
With acute malnutrition classifications appropriately treated with RUTF^d^	6	40(18,65)	7	70(38,92)	-30	6	50(23,77)	7	33(16,55)	17	47	0.44

^a^Difference-in-differences: the difference in the proportion between the intervention and comparison areas at end-line subtracted by the difference in intervention and comparison area at baseline

^b^Denominator: Baseline (intervention = 43, comparison = 25); End-line (intervention = 57, comparison = 39)

^c^Denominator: Baseline (intervention = 118, comparison = 82); End-line (intervention = 116, comparison = 110)

^d^Denominator: Baseline (intervention = 15, comparison = 10); End-line (intervention = 12, comparison = 21)

*CI* Confidence interval, *ORS* Oral rehydration solution, *RUTF* Ready-to-use therapeutic food

^†^*P*-value obtained from the logistic regression model for the difference-in-difference analysis (for the difference between intervention and comparison groups over time). *P*-value was adjusted for clustering and age

## Discussion

Our analysis did not reveal any evidence that the complex intervention targeting communities, health facilities and district health managers was associated with improved assessment, classification or treatment of sick children with suspected pneumonia, diarrhoea, or acute malnutrition by health extension workers. The intervention did not improve the proportion of sick children that were seen by health extension workers that were trained in iCCM, had regular supervision or attended clinical mentorship meetings. The proportion of children seen by HEWs that were trained and supervised declined in both groups at end-line whereas the proportion that were seen by HEWs who had attended a clinical mentorship increased, more in the intervention group.

The approach used in the assessment of pneumonia was counting breaths for one minute, and that of acute malnutrition was MUAC tape measurement. Dehydration assessment was measured using abdominal skin pinching and checking whether skin goes back slowly or very slowly. There was no association between intervention and change in appropriate assessment of pneumonia, diarrhoea or malnutrition. Dehydration assessment for children with a diarrheal complaint was relatively high in intervention areas at baseline and decreased at end-line. In contrast, dehydration assessment was low in comparison areas at baseline and showed a sizable increase at end-line. Similarly, at end-line pneumonia assessment had increased in the comparison area and declined in the intervention area. While, malnutrition assessment was declined in both areas at end-line, more in the comparison area. Though a significant difference was observed in dehydration assessment over time, there was insufficient evidence to say that the intervention was associated with the decline. The possible reasons for improper assessment might be the lack of equipment’s such as timer at the health posts in the study area, and the low capacity of the health extension workers to adhere to the clinical guidelines. Similar gaps in the assessment of sick children following the clinical algorithm were observed in a study done in Ethiopia and a multi-country study conducted in Namibia, Kenya, Tanzania and Uganda [38, 39].

The complex intervention did not improve the health extension worker’s classification of pneumonia and malnutrition. For pneumonia, eight out of ten children with reported cough were classified according to guidelines in both areas at baseline whereas at end-line this had decreased to approximately half. The correct classification of malnutrition was over 90% in intervention and comparison areas at the start and end of study. The lack of association between the intervention and correct classifications might partly be due to improper assessments caused by the low level of iCCM supplies in the study area health posts. At baseline, only one-fifth of the health posts had functional timer in intervention and comparison areas and this has declined at end-line. In contrast, almost all health posts in intervention area and all health posts in comparison area had MUAC tape at the start and end of the study [32].

The intervention did not improve treatment for pneumonia, diarrhea and malnutrition. The proportion of appropriate pneumonia treatment declined in both areas at end-line with a greater decline in the comparison areas. The appropriate treatment of acute malnutrition with therapeutic food increased in the intervention areas and declined in the comparison areas at the end of the study; however, the change over time was not associated with the intervention. The lack of association between the intervention and correct treatment might partly be due to the low level of iCCM medicines. The overall evaluation of the complex intervention revealed that greater than three-fourths of health posts in intervention area and comparison area had amoxicillin at baseline and this has declined at end-line, with greater reduction in intervention area. There was no change in the availability of amoxicillin in the study area over time. The availability of oral rehydration solution increased in both areas and zinc availability was high at baseline and end-line in both groups. The availability of ready-to-use therapeutic food was low and declined in both areas [32].

A study that assessed the health extension workers perceived context and preparedness of health posts in the same setting revealed that there was a low level of resources and preparedness of health posts to provide child health services [40]. Ensuring iCCM medicines availability, and training of health extension workers to monitor their stock of medicines is important [41, 42]. Without adequate commodities, healthcare providers cannot provide life-saving treatments to their communities and are at risk of losing community trust [43].

The lack of associations might be also related to the low knowledge of the health extension workers on the management of sick children including drug provisions. A study conducted in Ethiopia revealed that a considerable proportion of the health extension workers had poor knowledge of drug provision for childhood illnesses, and their knowledge of sick child management was associated with presence of continuous supervisions and refresher trainings [44]. Another study conducted on the sick children referral practices of the health extension workers reported that lack of knowledge of treatment guidelines and skills was hindrance to adherence of the clinical guidelines [45].

In the present study area, most of the sick children were assessed, classified and treated by health extension workers who utilized the iCCM chart booklet and the registration book. Although adherence to iCCM principles may increase the quality of services [46, 47], the health extension workers did not properly follow these guidelines. Improper assessments negatively influence subsequent classification and treatment of sick children, exposing children to missed opportunities of lifesaving interventions. A previous study reported that the complexity of the guidelines could hinder adherence to recommended actions [48]. Proper diagnosis and treatment of sick children are prerequisites for reducing under-five deaths in pneumonia, diarrhoea and severe acute malnutrition [43]. Many children’s lives can be saved with correct management of childhood diarrhoea [49, 50].

The OHEP intervention aimed to support the health extension workers by building their capacity to provide iCCM services. Previous research has shown positive relationships between capacity building interventions and aspects of quality of care provided by community health workers. Trained, supported and well-equipped community health workers can implement iCCM according to the guidelines [20, 51, 52]. However, there was no increase in iCCM-trained health extension workers that could be attributed to the intervention. Furthermore, iCCM training was provided to approximately eight out of ten health extension workers in both areas at baseline and end-line, implying that better coverage of training is necessary but not sufficient intervention to improve the quality of health care [32]. In addition to coverage, the duration and frequency of trainings, as well as the general conditions for health workers influence the quality of care that is provided. Earlier studies have also reported that in-service training is more effective when provided jointly with supervision and mentorship [53, 54]. An approach that combined training with facility-based practice, coaching and continuous supportive supervision and mentorship of community health workers has improved their skills and retention [52]. OHEP was intended to provide joint supervision by district health staff and biannual performance reviews and clinical mentoring meetings. However, supervision or clinical mentorship did not increase as a result of the intervention [32].

The process evaluation of the complex intervention has also indicated that the capacity building interventions had low coverage. Some of the intervention packages were introduced late and in some of the districts the packages of interventions were interrupted for months due to administrative issues. Only a fifth of the intervention activities had high fidelity of implementation in the first year [37]. This could potentially explain the lack of change in the quality of care provided by the health extension workers in the study area. The lack of associations between OHEP intervention and improved quality of care might also be related to the level of education of the health extension workers, and lack of in-kind incentives as reported from Ethiopia and other parts of the world [44, 55–57]. In the OHEP evaluation areas health extension workers with a lower level of training (level-3) were more common and only few of them were provided with an incentive accommodation. Intervention and comparison area health extension workers, however, were similar at baseline and end-line with respect to their level of education, service experience, and having accommodation.

The overall evaluation of OHEP intervention also revealed a lack of effect on care-seeking for common childhood illnesses in children 2–59 months’ age. The intervention had no effect on household participation in community engagement forums, nor on indicators of district accountability of child health services [32]. Another study based on the same survey data also revealed a lack of association between the intervention and correct classification of sick children 2–59 months by the health extension workers as compared to health officers who served as a gold standard [58].

The study clusters with health posts in intervention and comparison areas were selected for the evaluation of the intervention. However, we believe that study health posts and health extension workers were not substantially different from the average in the four study regions. Some health posts were excluded due to security reasons, and this might theoretically bias the findings. These excluded health posts were few and occurred in equal proportions in intervention and comparison areas. The intervention and comparison areas were similar in baseline characteristics [32]. Child age was an exception, but this was accounted for in the analyses. The study was based on observation of sick children’s consultations by health extension workers and observations were conducted by trained clinicians who also had received refresher training of iCCM. The observers silently followed and recorded the consultations [59]. The results may therefore potentially be influenced by a Hawthorne effect [60–62], implying that the daily management without observation could be even less correct. The outcome was measured at two points (baseline and end-line) and the trend or speed of changes of outcome in the absence of the program (time-varying differences) in both areas was not assessed. This might partly underestimate the effect of the complex intervention.

## Conclusion

We have shown that a complex intervention had no association with appropriate assessment and management by the health extension workers of children with suspected pneumonia, diarrhoea and acute malnutrition. The lack of association might be linked to a low coverage and poor implementation of the interventions. Our findings confirm earlier reports that training of health care providers without continued mentoring and support does not improve the quality of care. Therefore, such kind of community level complex intervention need adequate time and high coverage of implementation to bring change. It is critical to strengthen community-based programs with clinical mentorship, supportive supervision and supply of medicines and other essential commodities.

## Data Availability

Data from this study are co-owned by the participating institutions and stored in a depository at the Ethiopian Public Health Institute (EPHI). The use of these data is guided by a data sharing agreement that states that data will be made available upon reasonable request but are not publicly available during the period when Ph.D. students and other involved researchers are analyzing and reporting based on these data. Data can be accessed from the secretary of Data sharing committee of EPHI-LSHTM collaborative projects, Mrs. Martha Zeweldemariam; E-mail: martha.zeweldemariam@lshtm.ac.uk.
